# Golden Wedding Munificence

**Published:** 1906-12-01

**Authors:** 


### GOLDEN WEDDING MUNIFICENCE.

### £100,000 FOR RESEARCH AND CHARITY.

We have often had the pleasure to testify to the generous and large-minded consideration which Mr. H. L. Bischoffsheim has displayed towards philanthropic enterprise of the highest kind. Mr. and Mrs. Bischoffsheim have celebrated their golden wedding by distributing £100,000 with the idea to make others happy. The character of this golden-wedding gift will be seen from the following list:—

GENERAL CHARITIES.

| | £ |
|---|---|
| Imperial Cancer Research Association ... ... | 40,000 |
| King Edward's Hospital Fund ... ... ... | 10,000 |
| Tuberculosis Research in connection with the King Edward VII. Sanatorium ... ... | 10,000 |
| London Hospital ... ... ... ... ... | 2,500 |
| Middlesex Hospital ... ... ... ... | 2,500 |
| Charing Cross Hospital ... ... ... ... | 2,500 |
| Queen Charlotte's Lying-in Hospital ... ... | 2,500 |
| Great Ormond Street Children's Hospital ... | 2,500 |
| Metropolitan Provident Medical Association... | 2,500 |
| St. George's Hospital ... ... ... ... | 1,000 |
| Surgical Aid Society ... ... ... ... | 1,000 |
| Metropolitan Association for Befriending Young Servants ... ... ... ... ... | 1,000 |
| Metropolitan Convalescent Homes Association | 1,000 |
| Travellers' Aid Society ... ... ... ... | 500 |
| Soldiers' and Sailors' Help Society ... ... | 500 |

JEWISH INSTITUTIONS.

| | |
|---|---|
| Daneswood Sanatorium ... ... ... ... ... | 10,000 |
| Tudor House ... ... ... ... ... ... | 6,000 |
| Jewish Association ... ... ... ... ... | 1,000 |
| Jews' Free School ... ... ... ... ... | 1,000 |
| Jews' Infant School ... ... ... ... ... | 1,000 |
| Jewish Board of Guardians ... ... ... | 1,000 |

It is characteristic of Mr. Bischoffsheim that he should have had the wisdom and foresight to contribute the splendid sum of £40,000 to be devoted to Cancer Research. We confess to the assured belief that this money so given may prove more fruitful in benefit to humanity than it could ever have been had it been devoted to any other object. If wealthy people could once be made to realise that were an adequate sum made available for research purposes humanity might be virtually protected from the ravages of several of the most fatal diseases known to mankind, Mr. Bischoffsheim's action would find many imitators. His further gift of £10,000 to tuberculosis research proves to demonstration the thoroughness which underlies this munificence, as represented by the knowledge from which these gifts take their origin.

We have always felt that persons of wealth who possess wisdom and courage should distribute their charities during their life. Mr. and Mrs. Bischoffsheim evidently possess these great qualities. They are to be congratulated upon the form of their golden-wedding celebration, and everyone will wish them good health and many years in which to enjoy the happiness which by their gifts they hope to extend to others less fortunate than themselves.

